# Virulence Analysis of *Bacillus cereus* Isolated after Death of Preterm Neonates, Nice, France, 2013

**DOI:** 10.3201/eid2305.161788

**Published:** 2017-05

**Authors:** Romain Lotte, Anne-Laure Hérissé, Yasmina Berrouane, Laurène Lotte, Florence Casagrande, Luce Landraud, Sabine Herbin, Nalini Ramarao, Laurent Boyer, Raymond Ruimy

**Affiliations:** Centre Hospitalier Universitaire de Nice, Nice, France (R. Lotte, A.-L. Hérissé, Y. Berrouane, L. Lotte, F. Casagrande, L. Landraud, R. Ruimy);; Université de Nice Côte d’Azur, INSERM, C3M, Nice (R. Lotte, L. Lotte, L. Landraud, L. Boyer, R. Ruimy);; INSERM U1065, C3M, Team 6, Nice (R. Lotte, L. Boyer, R. Ruimy);; Food Safety Laboratory, Maison Alfort, France (S. Herbin);; Micalis Institute, INRA, AgroParisTech, Université Paris-Saclay, Jouy-en-Josas, France (N. Ramarao)

**Keywords:** Bacillus cereus, emerging pathogen, virulence, bacteria, environmental pathogen, neonates, Nice, France

## Abstract

After the deaths of 2 preterm neonates with *Bacillus cereus* systemic infection in the same intensive care unit, we investigated the pathogenic potential of this bacterium. Genetic and virulence analysis indicated the neonates were infected with 2 different strains with a virulence potential similar to environmental strains, indicating likely patient immune response failure.

*Bacillus cereus* is a gram-positive, spore-forming bacterium that is widespread in the environment. In adults, *B. cereus* is involved mainly in gastrointestinal infection and is the third most common cause of food poisoning (*1*). Rarely, this bacterium causes invasive or fatal infections in high-risk patients, such as immunocompromised adult patients and preterm neonates who have an immature immune system that is mostly restricted to innate immunity (*2*–*4*).

In 2013, two preterm infants with *B. cereus* infection died in the same intensive care unit. As part of the investigation of these deaths, we conducted genetic and virulence analyses of *B. cereus* strains from the patients and from the environment.

## The Study

In September 2013, tracheobronchial aspiration and blood cultures positive for *B. cereus* were obtained from 2 premature newborns hospitalized in the same intensive care unit. An unfavorable outcome led to the infants’ deaths despite an appropriate treatment with wide-spectrum antibiotic drugs.

The first premature infant was female, born at 27 weeks and 2 days of gestation, and weighed 880 g. An emergency cesarean delivery was performed because of the mother’s preeclampsia. The Apgar score at birth was 1-2-10, with bagging ventilation and intubation at 5 min after birth. No evidence of maternal–fetal transmission of infection was retrieved. On day 4, signs of infection were noted in the newborn, including respiratory distress, tachycardia, and a gray skin complexion. Investigations revealed elevated inflammatory markers (C-reactive protein level 88 mg/L). Empirical intravenous antimicrobial drug therapy (cefotaxime, gentamicin, and vancomycin) was started.

Tracheobronchial aspiration was performed and, a sample grew 10^6^ CFU/mL of *B. cereus* identified by matrix-assisted laser desorption/ionization time-of-flight mass spectrometry (MicroFlex LT; Bruker Daltonics, Billerica, MA, USA) (log score value of 2.07 matching with *B. cereus* reference strain DSM 31T, MALDI Biotyper v2.3). The blood culture remained sterile after 14 days. During her stay, the neonate had refractory hypoxemia due to a diffuse pulmonary lung parenchymal necrosis that required high-frequency ventilation and continuous thoracic drain. Despite an appropriate antimicrobial drug treatment (15 days of vancomycin followed by fluoroquinolone), the neonate had chronic hypoxemia and died at 26 days of age.

The second premature neonate, born 2 days after the first, was male, born at 29 weeks and 4 days of gestation, and weighed 1,480 g. A cesarean section was performed to enable the mother to start chemotherapy for a maternal malignancy, diagnosed at 26 weeks of gestation. The Apgar score at birth was 10. Physical examination indicated no sign of maternal or neonatal infection. On day 4, signs of infection were observed in the newborn, along with respiratory distress. The infant was reintubated, and antimicrobial drug therapy (cefotaxime, gentamicin, and vancomycin) was started.

**Figure 1:**
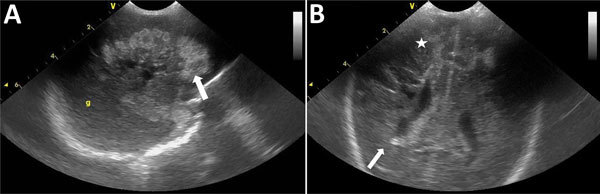
Standard echography cranial ultrasound of premature infant with *Bacillus cereus* sepsis, Nice, France, 2013. A) Left sagittal section showing large hemorrhagic hyperechogenic area of white material (white arrow). B) Frontal section showing right periventricular kystic hypoechogenic lesions (white arrow) with associated bilateral hemorrhagic hyperechogenic lesions (white star).

Blood cultures were positive after 9 hours, and subcultures grew with *B. cereus* (log score 2.02). Catheter cultures were positive and grew 10^6^ CFU/mL of *B. cereus* (log score 2.1). On day 5, despite appropriate care and sepsis control, the newborn showed severe neurologic impairment. Control cranial ultrasound revealed brain empyema, cerebral necrosis, and cranial hemorrhages (Figure 1). An unfavorable outcome led to the patient’s death at 8 days of age from multiple organ failure and cerebral abscesses.

**Figure 2:**
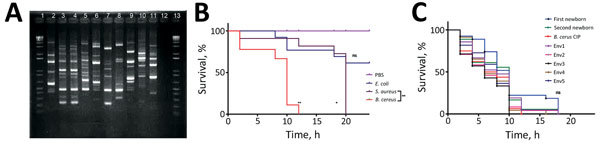
Genetic and virulence analyses of *Bacillus* spp. strains isolated from 2 preterm neonates with *B. cereus* infection and environmental sampling from intensive care unit, Nice, France, 2013. A) Molecular typing using M13-PCR methods, as described by Guinebretiere et al*.* (*5*). Lane 1, DNA ladder; lane 2, tracheobronchial fluid, first newborn; lane 3, blood culture, second newborn; lane 4, catheter, second newborn; lane 5, incubator surface, first newborn (Env1); lane 6, incubator surface, first newborn (Env2); lane 7, incubator surface, first newborn (Env3); lane 8, ultrasonographic probe (Env4); lane 9, bench surface used for bottle feeding (Env5); lane 10, incubator surface, second newborn (1); lane 11, incubator surface, second newborn (2); lane 12, negative control; lane 13, DNA ladder. B) Survival of flies infected with *Escherichia coli* CIP 102181, *Staphylococcus aureus* CIP 110856, and *B. cereus* CIP 66.24 T, compared with survival of control flies injected with phosphate-buffered saline (PBS). *p <0.01; **p <0.001; ns, not significant (all by Gehan-Breslow-Wilcoxon χ^2^ test). C) Survival of flies infected with the different strains of *B. cereus* tested. Env, environmental; ns, not significant by Gehan-Breslow-Wilcoxon χ^2^ test.

The hospital’s infection control team looked for environmental reservoirs as potential sources of contamination of the 2 newborns. Therefore, ventilation equipment, balloons used in manual ventilation, intravenous umbilical catheters, ultrasonic probes, linens (including towels and bedsheets), breast milk, and freeze-dried breast milk were collected and sent for microbiological analysis (Table 1). *B. cereus* cultures were positive for 5 environmental samples, including the surface of the incubator used for the first newborn (3 samples), ultrasonic probes (5 samples), and a bench surface used for bottle-feeding (5 samples). We compared all *B. cereus* strains, including those isolated from the 2 newborns, by using M13-PCR methods (*5*). This analysis revealed that the patients were infected by 2 different strains and that the environmental strains were different from strains isolated from patients. These data excluded a clonal transmission between the 2 patients and the hypothesis of a nosocomial outbreak caused by an emerging virulent strain (Figure 2, panel A). Nevertheless, a common source of infection for the 2 newborns by polyclonal strains cannot be excluded.

**Table 1:** Microbiological results of environmental sampling after deaths of 2 preterm neonates with *Bacillus cereus* infection, Nice, France, 2013*

Environmental site	No. positive/no. samples tested
Incubator, first newborn	3/7
Incubator, second newborn	0/7
Ultrasonographic probe	1/1
Bench surface used for bottle feeding	1/1
Control incubator 1	0/4
Control incubator 2	0/4
Control ultrasonographic probe	0/1
Heated humidifier for 2 control incubators	0/2
Wet bulb for 2 control incubators	0/2
Heating ramp for control incubator 1	0/1
Control incubator mattresses	0/2
Babies’ sheets	0/1
Towels	0/1
Sterile field	0/1
Ultrasound gel dispenser bottles	0/2
Used ultrasound gel	0/1
Air filtration/ventilation equipment	0/1
Air flow sensor for ventilator	0/1
Heating unit for ventilator	0/1
Food refrigerator	0/1
Various cutlery	0/2

We screened the isolated strains for *B. cereus* main virulence factor genes hemolysin BL, nonhemolytic enterotoxin, cytotoxin K, and hemolysin II (Table 2) by using PCR and toxin production assay methods (*6*–*9*). Both patient and environmental isolates produced toxins. We further assessed the virulence potential using an in vivo model of *Drosophila melanogaster* infection. To validate the capacity of this model to detect the virulence of various bacterial strains, we first infected wild-type flies with *Escherichia coli* CIP 102181, *Staphylococcus aureus* CIP 110856, and *B. cereus* CIP 66.24T. We grew bacteria in Luria-Bertani broth overnight at 37°C and subcultured them up to an optical density of 0.8 at 600 nm. We dipped a tungsten needle into an equal volume of bacterial suspension or phosphate-buffered saline (control) and used it to prick 20–30 adult male flies (*10*). All flies infected with *S. aureus* and *B. cereus* died after 20 and 12 hours, respectively. Flies infected with *E. coli* displayed a survival rate similar to that of control flies, enabling us to validate *D. melanogaster* as a model for evaluating the strains’ virulence potential (Figure 2, panel B). 

**Table 2:** Virulence factor analysis of strain characteristics of bacteriologic samples obtained after deaths of 2 preterm neonates with *Bacillus cereus* infection, Nice, France, 2013*

Tested strain	Source of sample	Gene				Genotype group (%)	Nhe production inde)†	Hbl detection limit
		*cytK1 *	*cytK2 *	*ces *	*hlyII *			
First newborn	Tracheobronchial aspiration	–	+	–	–	III (99.72)	+++	–
Second newborn	Blood culture	–	+	–	–	IV (100)	+	1/64
Second newborn	Catheter	–	+	–	–	IV (100)	+	1/64
Environmental isolate 1	Incubator surface, first newborn	–	–	–	–	II (97.71)	++	–
Environmental isolate 2	Incubator surface, first newborn	–	+	–	–	III (99.72)	+++	–
Environmental isolate 3	Incubator surface, first newborn	–	+	–	+	IV (100)	+++	1/64
Environmental isolate 4	Ultrasonographic probe	–	+	–	–	IV (100)	+++	1/32
Environmental isolate 5	Bench surface used for bottle feeding	–	–	–	–	III (100)	+++	–

*Hbl, hemolytic BL toxin; Nhe, nonhemolytic enterotoxin; +, positive; –, negative. †Nhe production level: +, low; ++ moderate; +++, high.

We used the same protocol to compare all *B. cereus* strains. We found no statistical difference in survival between flies infected with the different *B. cereus* strains, including *B. cereus* CIP 66.24T (Figure 2, panel C). These data correlate with the absence of a specific virulence signature for those strains (Table 2).

## Conclusions 

Considering the fatal outcome of the 2 infections despite appropriate antimicrobial drug therapy, we addressed the question of a high virulence potential of the patients’ *B. cereus* strains by testing for the presence of virulence factor genes and expression levels. We found a similar virulence factor profile in the patients and in the environmental strains. This profile suggested that the outcome of the infection was probably not linked to the virulence potential of the strains. 

We then used *D. melanogaster* as an infection model. We chose this model because flies rely only on innate immunity to survive infections, similar to preterm newborns, who have immature immune systems. We found that all isolated *B. cereus* strains (patient and environmental strains) displayed a similar killing potential, suggesting that the fatal outcome in both newborns was due not to the emergence of a hypervirulent strain but rather to a similar pathogenic potential for all *B. cereus* strains toward at-risk patients.

Given that *B. cereus* is ubiquitous in the environment and potentially fatal in preterm neonates, it appears critical to determine how these 2 neonates were infected and why they died, as well as why other preterm neonates hospitalized concurrently in the same room of the intensive care unit remained uninfected. Further investigations would be necessary to determine whether the deaths were a consequence of an innate immune defect, a high bacterial load at time of contamination, or a combination of both parameters.

Our study, along with previous ones (*3**,**4**,**11*–*13*), reinforces the idea that *B. cereus* is an underestimated emerging pathogen that can be involved in fatal healthcare-associated infections in premature newborns. Our results indicate that all *B. cereus* strains display potentially pathogenic properties toward at-risk patients. Considering that *B. cereus* is ubiquitous in the environment, it is essential to emphasize the necessity of strict hygiene measures and protocols to prevent bacterial transmission. Evaluating an immune response capacity in at-risk patients must be considered to avoid a fatal outcome from *B. cereus* infection.
